# Abnormal presentation of ascending cholangitis

**DOI:** 10.1002/ccr3.1357

**Published:** 2018-04-06

**Authors:** Mark Scaife, Ryan Abegglen, Christina Vila, Kurt Stahlfeld

**Affiliations:** ^1^ Department of Surgery UPMC Mercy 1400 Locust St Pittsburgh 15219 Pennsylvania

**Keywords:** Ascending cholangitis, endoscopic retrieval, retained shunt, shunt fragment, ventricular shunt

## Abstract

Ventriculo‐peritoneal, ‐gallbladder, ‐pleural, ‐atrial, and ‐jugular shunts are all viable options in the treatment of hydrocephalus 1, 2, 3. Retrieval of these catheters can often be very difficult and may be unsuccessful or incomplete. Retained catheters can result in unforeseen and unexpected complications.

A female in her 20s with a history of spina bifida, hydrocephalus, and placement of multiple ventriculo‐peritoneal/pleural/gallbladder/atrial shunts was referred for severe right upper quadrant pain, fever, and hypotension. Several years earlier, one of her multiple shunts reportedly fractured during explantation and the distal fragment was not retrieved, subsequently migrating into the CBD and lodging at the ampulla (Fig. 1A). Our patient underwent ERCP (Fig. 1B), sphincterotomy, and extraction of a long segment of a ventricular drainage catheter (Fig. 1C) and subsequently recovered from her episode of acute ascending cholangitis with medical therapy 4, 5, 6. Several months following discharge, a laparoscopic converted to open cholecystectomy retrieved an additional shunt fragment that remained in her gallbladder. She subsequently made an uneventful recovery.

**Figure 1:**
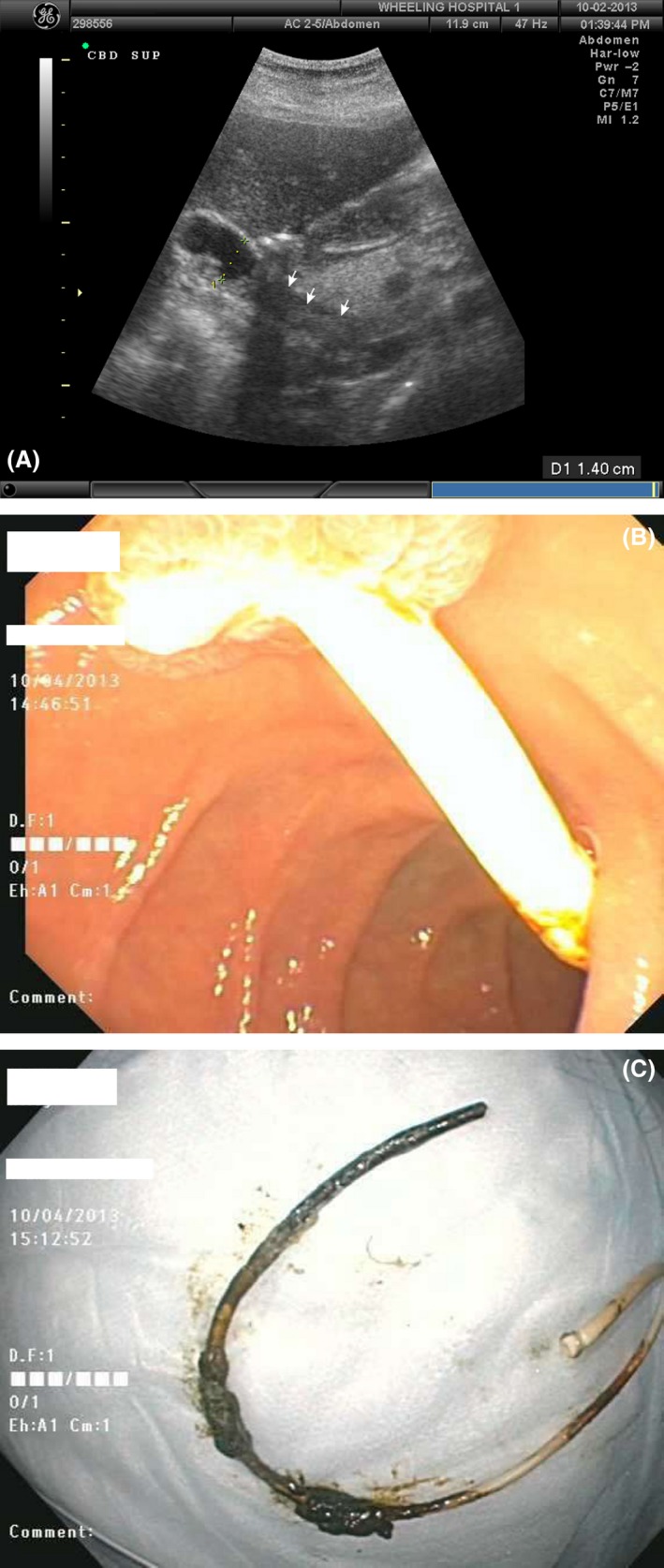
(A) Ultrasound showing a foreign object retained within the common bile duct. (B) ERCP image demonstrating retained shunt lodged within the ampulla. (C) Long segment of retained ventriculo‐gallbladder shunt following sphincterotomy and endoscopic retrieval.

## Authorship

MS: reviewed the patient, performed the literature review, and wrote the manuscript. CV and RA: reviewed the patient, and reviewed and edited the manuscript. KS: supervised the research project, reviewed the patient, and reviewed and edited the manuscript.

## Conflict of Interest

None declared.
